# TIPE2 negatively regulates *mycoplasma pneumonia*-triggered immune response via MAPK signaling pathway

**DOI:** 10.1038/s41598-017-13825-y

**Published:** 2017-10-17

**Authors:** Yuanyuan Zhang, Shufen Mei, Yunlian Zhou, Dehua Yang, Ting Pan, Zhimin Chen, Qingqing Wang

**Affiliations:** 1 The Children’s Hospital of Zhejiang University School of Medicine, Hangzhou, 310051 P. R. China; 2 Institute of Immunology, Zhejiang University, Hangzhou, 310058 P. R. China; 3 Departement of Pediatrics, Red Cross Hospital of Hangzhou, Hangzhou, 310003 P. R. China

## Abstract

Excessive immune responses played an important role in pathophysiology of *mycoplasma pneumonia* (MP) infection. Tumor necrosis factor-α-induced protein 8-like 2 (TIPE2) is a negative regulator of immune response. This study investigated the expression change of TIPE2 and its role in immune defense against MP infection, as well as the underlying mechanisms. Expressions of TIPE2 both in patients and in macrophages *in vitro* after MP infection were measured. We further studied cytokine production and mitogen-activated protein kinase (MAPK) signaling function in macrophages with interfered expression of TIPE2 upon MP infection. A significant decrease of TIPE2 mRNA expression was observed in peripheral blood mononuclear cells (PBMCs) from MP patients, which was correlated with the severity of infection. Accordingly we found down-regulation of TIPE2 expression in macrophages after MP infection. *In vitro* study further suggested that TIPE2 jeopardized inflammatory cytokine production trigged by MP infection via inhibiting MAPK signaling pathway. These findings provided evidences of the novel function of TIPE2 in anti-MP immunity and its possible clinical utility related clinical significance.

## Introduction

*Mycoplasma pneumoniae* (MP), an atypical bacterium, is a common pathogenetic organism of respiratory infection in children^1,2^. Prior studies showed that MP might account for as many as 40% of community-acquired pneumonia (CAP) cases and 18% of these patients require hospitalization^3^. Although *Mycoplasma pneumoniae* pneumonia (MPP) is usually self-limited, it may cause various pulmonary and extra-pulmonary complications sometimes^4^ and develop into refractory *Mycoplasma pneumoniae* pneumonia (RMPP)^5,6^ or even a life-threatening pneumonia^7,8^.

The pathophysiological mechanisms underlying MP infection still remain elusive, but excessive immune response was referred to as one contributing route in pathogenesis of MP infection^9^. Excessive immune response is believed to cause inflammatory diseases and autoimmunity. Recently, some studies reported that immune cells, such as macrophage, recognized lipoprotein or lipid peptide derived from MP through pattern recognition receptors (PRRs), and activated downstream signaling pathways to initiate innate immune response through induction of numerous cytokines (i.e., IL-1β, TNF-α, IL-6)^10–15^. Thus, the host immune system needs to balance activation and resolution of the immune response.

Tumor necrosis factor (TNF)-α-induced protein 8-like 2 (TIPE2, or TNFAIP8L2) shares considerable sequence homology with members of the TNF-α-inducible protein 8 (TNFAIP8) family which are thought to regulate cellular and immune homeostasis^16^. TIPE2 is preferentially expressed in lymphoid tissues, macrophages and lymphocytes^16^ and is recently identified as a negative regulator of innate and adaptive immunity. TIPE2 deficiency in mice leads to fetal inflammatory diseases, and abnormal expression of TIPE2 in humans was found to be associated with infectious diseases and autoimmune disorders, such as hepatitis B, systemic lupus erythematosus, asthma, and experimental stroke^17–20^. However, to date, it is unclear whether TIPE2 plays a role in regulation of immunity against MP infection.

Considering normal expression of TIPE2 gene is essential to maintain the immune homeostasis, we hypothesized that the TIPE2 gene might be involved in the immune response triggered by MP. Therefore, in this study, we first investigated the expression of TIPE2 both *in vivo* (in patients with MPP) *and vitro* (in macrophages after MP infection), and then identify the potential effects of TIPE2 on MP-triggered immune response *in vitro*.

## Results

### MP infection induces the decrease of TIPE2 expression in patients

**Figure 1:**
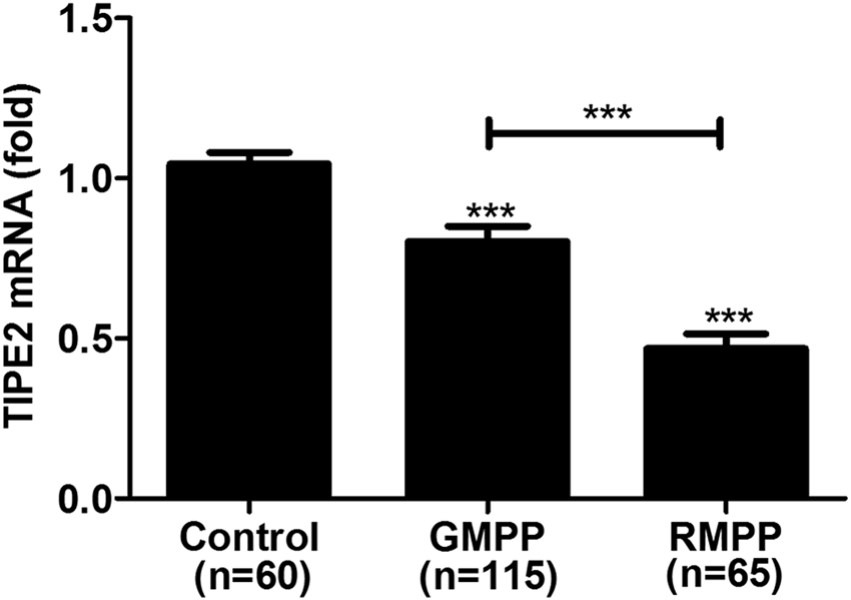
The expression levels of TIPE2 in PBMCs of MPP children and healthy controls. PBMCs were collected from 180 MPP children (115 cases of GMPP and 65 cases of RMPP) and 60 healthy controls, and TIPE2 mRNA levels were measured by real-time quantitative PCR. The levels of TIPE2 mRNA in PBMCs of MPP children were significantly lower, especially in RMPP children than those of the healthy controls (P < 0.05). Data are the mean ± SD. ***P < 0.001.

To investigate whether TIPE2 played a role in human defense against MP infection, we collected the peripheral blood samples from 180 pediatric MPP cases admitted to our hospital, and 60 healthy children as the control. According to the severity of clinical manifestation, MPP patients were divided to two groups: 115 patients in the GMPP group and 65 patients in the RMPP group. We detected TIPE2 mRNA expression in PBMCs in all cases from GMPP, RMPP and control group. The expression of TIPE2 mRNA in PBMCs was significantly decreased in MP patient groups than healthy controls, and was much lower in refractory cases (Fig. 1), indicating that the expression of TIPE2 in PBMCs might have a correlation with the anti-MP immune response of the host.

### Down expression of TIPE2 after MP infection *in vitro*

**Figure 2:**
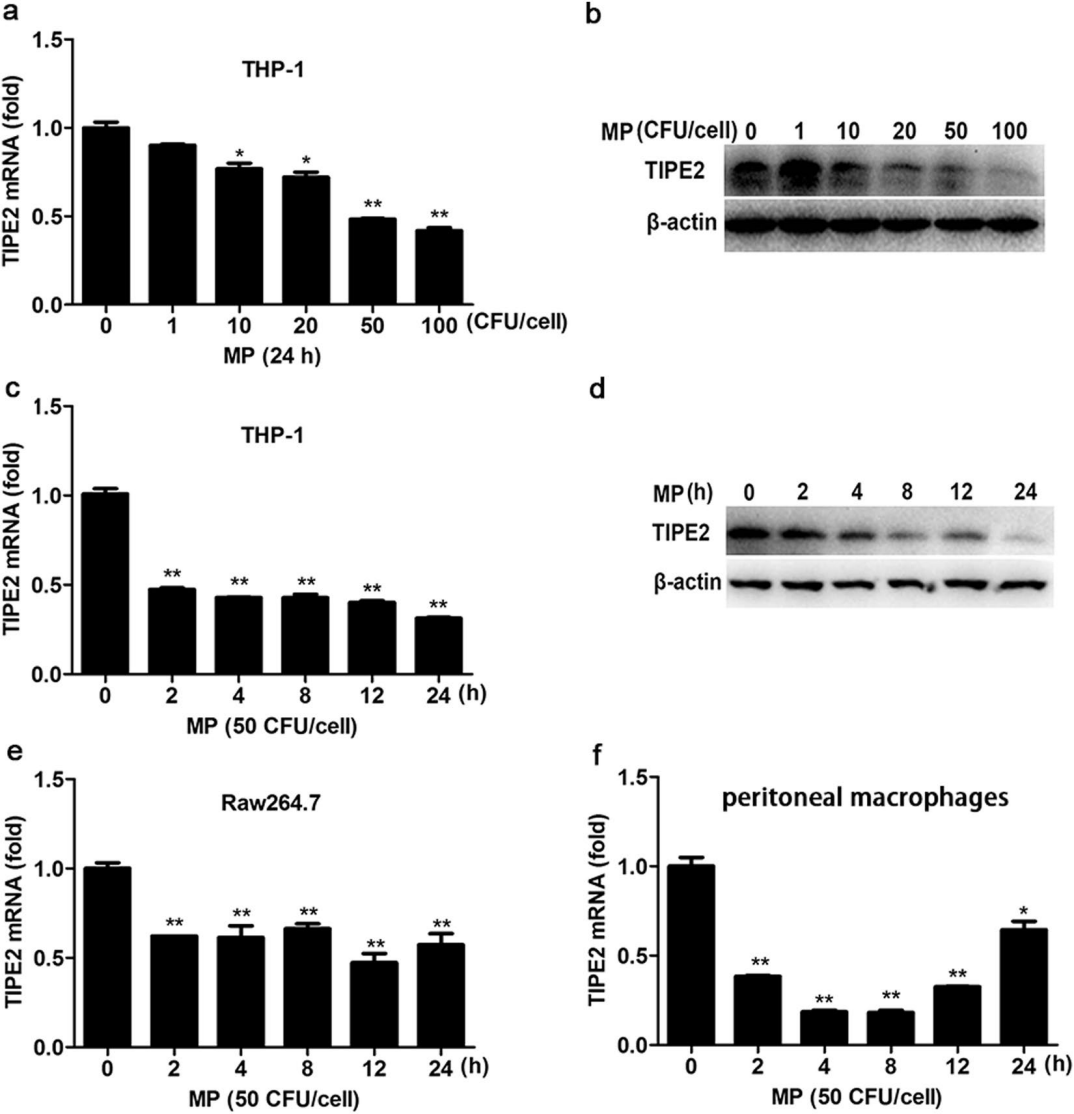
Down-regulation of TIPE2 expression in macrophages after MP infection. THP-1 cells were infected with different concentrations of MP for 24 h. The expression of TIPE2 was determined by qPCR (**a**) and Western-blot (**b**). THP-1 cells were infected with MP (50 CFU/cell) for indicated times. The expression of TIPE2 was measured by q-PCR (**c**) and Western-blot (**d**). Raw264.7 cells (**e**) and mouse peritoneal macrophages (**f**) were infected with MP (50 CFU/cell) for indicated times. The expression of TIPE2 was measured by q-PCR. Data are the mean ± S.D. (n = 3) of three independent experiments. *P < 0.05, **P < 0.01.

To further confirm the potential role of TIPE2 in the regulation of innate anti-MP response, we selected several cell types (THP-1 cells, RAW264.7 cells and primary peritoneal macrophages expressing TIPE2 to analyze the change in TIPE2 expression after MP infection via Q-PCR and/or Western-blot. In THP-1 cells, TIPE2 expression was inhibited by MP in a dose-dependent manner (Fig. 2a,b), featured by an significant decrease at 2 h post-infection and persisted low-expression at least 24 h after MP infection (Fig. 2c,d). In Raw264.7 cells (Fig. 2e) and primary peritoneal macrophages (Fig. 2f) down-regulation for TIPE2 was also observed after infection of MP. These results suggested that TIPE2 might function as a regulator of MP-associated immune response in macrophages.

### TIPE2 negatively regulated MP-triggered inflammatory cytokine production *in vitro*

**Figure 3:**
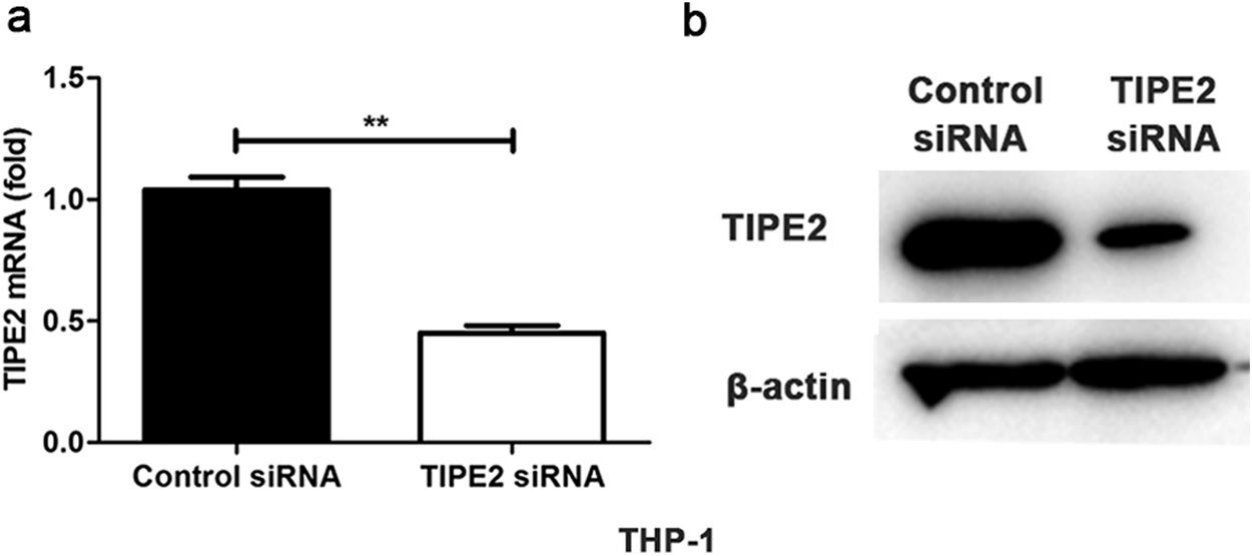
Silencing of endogenous TIPE2 expression by siRNA in THP-1 cells. After stimulating with 100 nM PMA, THP-1 cells were transfected with control siRNA or TIPE2 siRNA, as indicated at a final concentration of 20 nM. After 48 hr, TIPE2 expression was measured by qPCR (**a**) and Western-blot (**b**).

**Figure 4:**
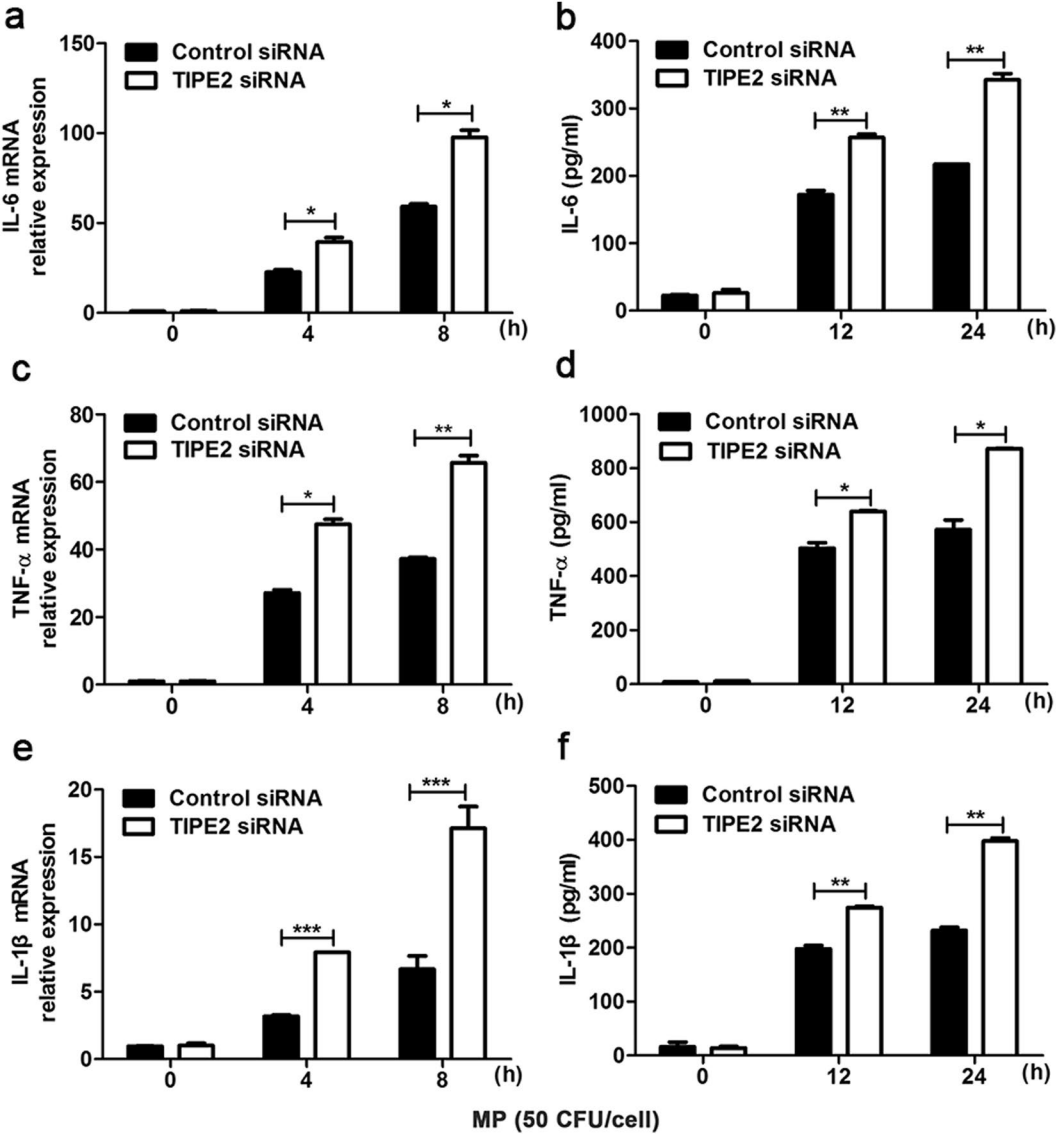
TIPE2 negatively regulated MP-triggered inflammatory cytokines production in macrophages. After stimulating with 100 nM PMA, THP-1 cells were transfected with control siRNA (NC) or TIPE2 siRNA, as indicated at a final concentration of 20 nM. 48 h later, cells were stimulated with or without MP (50 CFU/cell). IL-6 (**a**), TNF-α (**c**), IL-1β (**e**) mRNA levels were measured at 4 h and 8 h by qPCR after stimulation. Supernatants were collected after 12 h and 24 h to measure IL-6 (**b**), TNF-α (**d**), IL-1β (**f**) by ELISA. Data are the mean ± S.D. (n = 3) of three independent experiments. *P < 0.05, **P < 0.01, ***P < 0.001.

MP has been demonstrated to interact with macrophages and induce inflammatory cytokine production, such as IL-6, TNF-α and IL-1β by these cells^21^. To further identify whether MP-induced decrease of TIPE2 expression could affect MP-triggered response in macrophages, we investigated the relationship between TIPE2 expression and inflammatory cytokine production after MP challenge via Q-PCR and ELISA. As shown in Fig. 3a,b, TIPE2 gene in THP-1 cells was silenced by siRNA transfection. Meanwhile, we found that IL-6 production was extremely enhanced after MP infection at both mRNA and protein levels (Fig. 4a,b). Also, TNF-α and IL-1β expressions were up-regulated in TIPE2 silenced THP-1 cells infected with MP (Fig. 4c–f). Taken together, these results demonstrate that TIPE2 negatively regulates MP-triggered inflammatory cytokine (such as IL-6, TNF-α and IL-1β) production in macrophages.

### Anti-MP function of TIPE2 is mainly through MAPK signaling pathway

**Figure 5:**
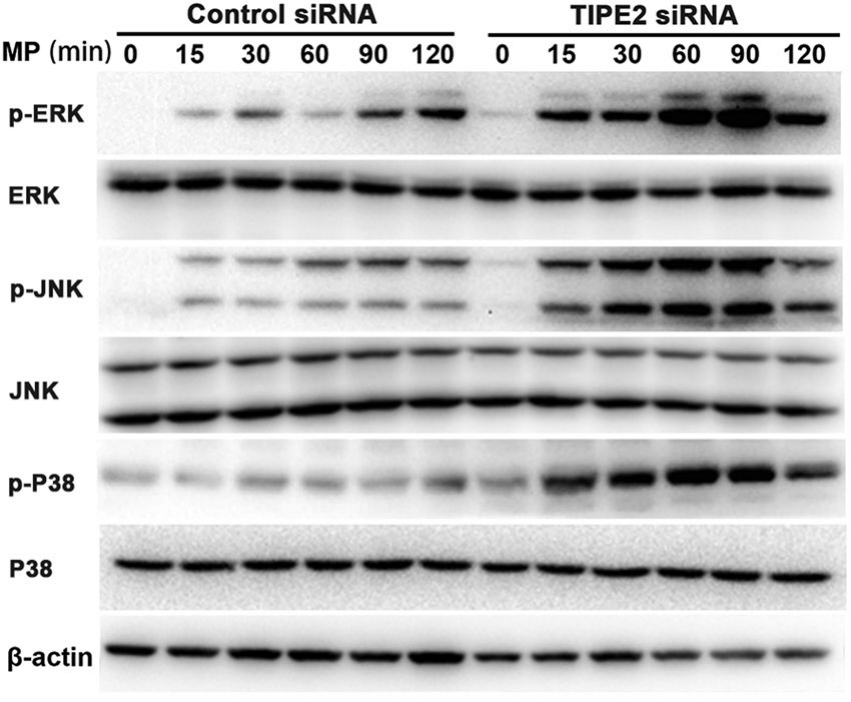
TIPE2 negatively regulated MP induced MAPK signaling pathway activation in macrophages. After stimulating with 100 nM PMA, THP-1 cells were transfected with control siRNA or TIPE2 siRNA, as indicated at a final concentration of 20 nM. 48 h later, cells were stimulated with MP (50 CFU/cell) for different time periods as indicated. Cell lysates were analyzed by Western-blot analysis using p-ERK1/2, p-JNK, and p-P38. β-actin was used as the loading control. Data shown are representative of one experiment out of three.

**Figure 6:**
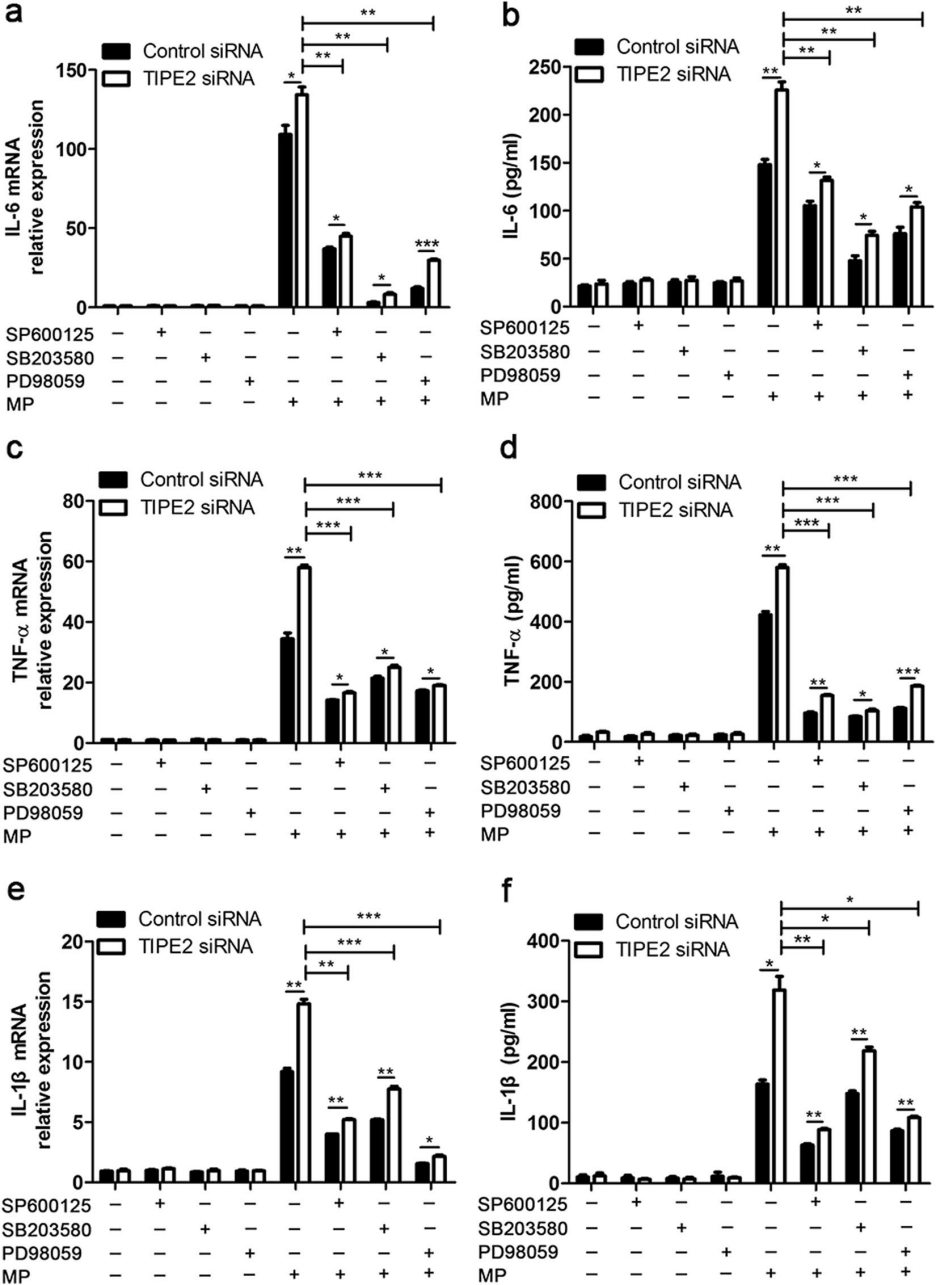
TIPE2 inhibits MP-triggered inflammatory cytokines production via inhibiting MAPK activation. After stimulating with 100 nM PMA, THP-1 cells were transfected with control siRNA (NC) or TIPE2 siRNA, as indicated at a final concentration of 20 nM. The transfectants of THP-1 cells were preincubated with 30 μM SP600125, SB203580 or PD98059 for 3 h before being stimulated with 50 CFU/cell MP. IL-6 (**a**), TNF-α (**c**), IL-1β (**e**) mRNA levels were measured at 8 h by qPCR after stimulation. Supernatants were collected after 12 h to measure IL-6 (**b**), TNF-α (**d**), IL-1β (**f**) by ELISA. Data are the mean ± S.D. (n = 3) of three independent experiments. *P < 0.05, **P < 0.01, ***P < 0.001.

It has recently been demonstrated that MAPK pathway is involved in MP-mediated cytokine induction^22^.So we further investigated the function of downstream MAPK signal pathway in macrophages infected by MP with interfered TIPE2 expression. We found that low expression of TIPE2 did significantly increase phosphorylation of p38, ERK, JNK after MP challenge (Fig. 5)(the original blots were shown in supplementary Fig. 5). These data indicated that TIPE2 inhibited MAPK activation in macrophages. To determine whether the above TIPE2-mediated anti-MP function requires the MAPK activation, we preincubated the THP-1 cells with MAPK inhibitors (30 μM JNK inhibitor SP600125, P38 inhibitor SB203580 or ERK inhibitor PD98059) for 3 h, and then stimulated the cells with MP (50 CFU/cell). As demonstrated in Fig. 6, SP600125, SB203580 or PD98059 partially reversed the inhibitory effect of TIPE2 on MP-induced inflammatory cytokine production in THP-1 cells (Fig. 6). Collectively, these findings suggest that negative regulation of MP-triggered cytokines production by TIPE2 might be mediated through MAPK signaling.

## Discussion

MP is one of the most prevalent pathogens causing CAP in children. MPP is usually a benign self-limited disease, but cases of RMPP, which display clinical and radiological progression after macrolide therapy for 7 days or longer, are reported increasingly^5,6^. The reasons why RMPP occurred remain unclear, but it is widely accepted that immunological response plays an important role in it^6,9,23,24^. TIPE2 is recently described as a novel gene independently required for maintaining immune homeostasis, which is constitutively expressed in various stages of macrophage, B and T lymphocytes, and it belongs to TNFAIP8 family^16^. This is the first study to characterize TIPE2’s role in MP-triggered immune response and possible connection with clinical features in pediatric patients.

In this study, we first found that the expression of TIPE2 is associated with MP infection. Our results showed that the expression of TIPE2 mRNA from PBMC decreased in MPP patients compared to healthy children. Furthermore, the expression of TIPE2 mRNA from PBMC in RMPP patients decreased more significantly indicating a negative correlation between the expression of TIPE2 and clinical severity of MPP. Interestingly, we also detected the expression of TIPE2 in macrophage decreased significantly after MP stimulation *in vitro*. Therefore, the decreased expression of TIPE2 upon MP infection suggested that TIPE2 might contribute to the regulation of MP-associated immune response.

It is well known that inflammatory cytokines play a critical role in MP infection. Several studies indicated that inflammatory response in lung tissue responsible for MP disease is associated with an increased and prolonged expression of IL-6, TNF-α, IL-1β and IL-17^21,25,26^. Clinical research also showed that serum levels of inflammatory cytokines, such as IL-6, IL-10, TNF-α, and interferon gamma (IFN-γ) significantly increased in MPP patients than those in control^27,28^. Our previous study also showed that levels of IL-6, IL-10, and IFN-γ in RMPP group were significantly higher than those in GMPP group^4^. Sun *et al*. demonstrated that TIPE2 in mouse macrophages inhibit the secretion of some inflammatory cytokines^16^. Therefore, it is imperative to know whether TIPE2 expression is associated with the production of inflammatory cytokines caused by MP infection. Our data showed that silence of TIPE2 expression in macrophage prompted the secretion inflammatory cytokines (such as IL-6, TNF-α and IL-1β) to MP stimulation. These results indicated that TIPE2 could negatively regulated MP-triggered inflammatory cytokine production in macrophages.

Previous studies showed that MP could activate the MAPK signaling by phosphorylation of ERK1/2, p38 and JNK proteins, leading to a large-amount production of inflammatory cytokines^12^. In this study, we showed phosphorylation of ERK, p38, and JNK were increased in TIPE2-silenced macrophages with MP stimulation. Furthermore, the MAPK inhibitors, including JNK inhibitor, P38 inhibitor and ERK inhibitor could partially reverse the negative role of TIPE2 on MP-triggered cytokines production in THP-1 cells. Therefore, we postulated that the down-regulation of TIPE2 expression after MP infection might be responsible or partially responsible for the elevated levels of inflammatory cytokines, possibly via APK signaling pathway. However, the detailed molecular mechanisms by which TIPE2 inhibit intracellular signaling pathways remain to be further investigated.

In conclusion, our data indicated that TIPE2 expressed in macrophages might play a negative role in MP triggered immune response via inhibiting MAPK signaling pathway. This finding might not only advance our understanding of the mechanisms of MP infection, but also lead to the development of TIPE2-based strategies for treatment.

## Materials and Methods

### Human subjects and specimens

A total of 130 peripheral blood samples were collected from children with *Mycoplasma pneumoniae* pneumonia (MPP) hospitalized at the Children’s Hospital, Zhejiang University School of Medicine, China, from January 1, 2011 to December 31, 2014. The diagnostic criteria for pneumonia were according to symptoms and signs, such as fever, cough, abnormal lung auscultation and abnormal chest X-rays^5^. The diagnosis of MP infection was based on the positive results for serologic testing (MP IgM positive and antibody titer ≥ 1:160) combined with positive results for MP polymerase chain reaction (PCR) tests of nasopharyngeal aspirate/swab. RMPP was defined as prolonged fever and clinical and radiological deterioration after azithromycin treatment for 7 days or more^5,6^. According to disease severity, MPP patients were divided into two groups: general MPP (GMPP) and RMPP. A total of 60 healthy blood donors were used as controls. After collecting peripheral blood samples from patients and controls, peripheral blood mononuclear cells (PBMCs) were separated by density gradient centrifugation according to the manufacturer’s protocols.

### Mice and reagents

C57BL/6 mice (6-8wks old) were purchased from SIPPR-BK Experimental Animal Ltd Co. (Shanghai, China). All experiments and animal care were performed according to protocols approved by the Zhejiang University Institutional Animal Care and Use Committee. TIPE2 small interfering RNA (siRNA) and control siRNA were from GenePharma (Shanghai, China). PMA were from Sigma (Sigma-Aldrich LLC, USA). Abs specific to β-actin, and HRP-coupled secondary Abs were from Santa Cruz Biotechnology (Santa Cruz, CA). Abs specific to ERK, JNK, P38, phosphorylated ERK, phosphorylated JNK, and phosphorylated P38 were from Cell Signaling Technology (Danvers, MA). Abs specific to TIPE2 was from Abcam (Cambridge, MA). The JNK inhibitor SP600125, the P38 inhibitor SB203580 and the ERK inhibitor PD98059 were obtained from Selleck (Shanghai, China).

### MP culture, preparation and infection

Mp (strain M129-B7, ATCC 29342) was grown in SP-4 broth at 37 °C and 5% CO_2_. For preparation, MP were harvested by centrifugation (10000 × g at 4 °C for 20 min), washed and resuspended in PBS (pH 7.4) to yield 1 × 10^8^ CFU/ml. THP-1 cells were infected with MP (1, 10, 20, 50, 100 CFU/ml) for varying amounts of time (0, 2, 4, 8, 12, 24 h).

### Cell culture and transfection

THP-1 cell line was obtained from American Type Culture Collection (Manassas, VA, USA) and cultured in RPMI1640 containing 10% fetal calf serum (FBS). RAW264.7 cell line was obtained from American Type Culture Collection (Manassas, VA) and cultured in DMEM containing 10% FBS. Murine primary macrophages were obtained and cultured as previous described^29^. Human monocytes were prepared from PBMC by Ficoll/Hypaque density gradient centrifugation (Pharmacia LKB, Uppsala, Sweden). 5 × 10^5^ THP-1 cells were seeded into 24-well plates and incubated with 100 nM PMA for 48 h. 0.5 ml containing 5 × 10^5^ cells was seeded into each well of 24-well plates and incubated with 100 nM PMA for 48 h, and then when THP-1 cells were induced to macrophage, they were transfected with siRNAs using INTERFERin (Polyplus-transfection), according to the manufacturer’s instructions.

### RNA interference

The TIPE2-specific siRNA-419 was 5′-CCAUGACGGCACUUAGCUUTT-3′ (sense) and 5′-AAGCUAAGUGCCGUCAUGGTT-3′ (antisense). The TIPE2-specific siRNA-694 was 5′-GCACAUUCCACCUUGACAATT-3′ (sense) and 5′-UUGUCAAGGUGGAAUGUGCTT-3′ (antisense). The TIPE2-specific siRNA-497 was 5′-GGGAUGUGCUGCUAGAGUUTT-3′ (sense) and 5′-AACUCUAGCAGCACAUCCCTT-3′ (antisense). The control siRNA sequences were 5′-UUCUCCGAACGUGUCACGUTT-3′ (sense) and 5′-ACGUGACACGUUCGGAGAATT-3′ (antisense). siRNA duplexes were transfected into THP-1 cell at a final concentration of 30 nM.

### RNA isolation and real-time quantitative PCR (qPCR)

Total RNA was extracted with TRIzol (Invitrogen). Real-time quantitative PCR, using SYBR Green detection chemistry, was performed on a 7500 Real-Time PCR System (Applied Biosystems) as we previously described^30^. For human TIPE2, the qPCR primers were: 5′-GGAACATCCAAGGCAAGACTG-3′ (forward) and 5′- AGCACCTCACTGCTTGTCTCATC-3′ (reverse). For human β-actin, the qPCR primers were: 5′-CATGTACGTTGCTATCCAGGC-3′ (forward) and 5′-CTCCTTAATGTCACGCACGAT-3′ (reverse). For mouse TIPE2, the qPCR primers were: 5′-TCTCAGAAACATCCAAGGCC-3′ (forward) and 5′-TTTGAGCTGAAGGACTCCATG-3′ (reverse). For mouse β-actin, the qPCR primers were: 5′-AGTGTGACGTTGACATCCGT-3′ (forward) and 5′- GCAGCTCAGTAACAGTCCGC -3′ (reverse). For human TNF-α, the qPCR primers were: 5′-TGGCCCAGGCAGTCAGA-3′ (forward) and 5′-GGTTTGCTACAACATGGGCTACA-3′ (reverse). For human IL-6, the primers were: 5′-AGCCCTGAGAAAGGAGACATGTA-3′ (forward) and 5′-GGAGTGGTATCCTCTGTGAAGTCT-3′ (reverse). For human IL-1β, the primers were: 5′-GCCCTAAACAGATGAAGTGCTC-3′ (forward) and 5′-GAACCAGCATCTTCCTCAG-3′ (reverse). Data was normalized by the level of β-actin expression in each sample described above.

### Western-blot

The cells were washed twice with cold PBS and lysed with cell lysis buffer (Cell Signaling Technology) supplemented with protease inhibitor mixture (Beyotime). Protein concentrations of the cell lysis extracts were measured with BCA assay (Pierce, Rockford, IL) and equalized with the extraction reagent. Equal amount of the extracts were loaded and subjected to SDS/PAGE gel, transferred into PVDF membrane, and blotted as we previously described^31^.

### Detection of cytokine production

Into each well of 24-well plates, 0.5 ml containing 5 × 10^5^ THP-1 cells were seeded and incubated with 100 nM PMA for 48 h, and then transfected as described above. After 48 h, the cells were infected with MP for indicated time periods. The concentrations of TNF-α, IL-6 and IL-1β in culture supernatants were measured with ELISA kits (eBioscience) according to the manufacturer’s protocols.

### Approvals

All experiments were performed following the relevant guidelines and regulations of the Children’s Hospital, Zhejiang University School of Medicine. The methods were carried out in accordance with the approved guidelines. The study was approved by the ethics committee of the Children’s Hospital, Zhejiang University School of Medicine. Written informed consent was obtained from at least one guardian of each patient before enrollment.

### Statistical analysis

All experiments were repeated three times. The significance of difference between groups was determined by two-tailed Student’s *t* test and two-way ANOVA test. P values of less than 0.05 were considered statistically significant.

## Electronic supplementary material

Supplementary Information File
