# Supplementary material for: TIPE2 negatively regulates mycoplasma pneumonia-triggered immune response via MAPK signaling pathway
Source: Sci Rep. 2017 Oct 17;7:13319. doi: 10.1038/s41598-017-13825-y (PMC5645323; doi:10.1038/s41598-017-13825-y)

TIPE2 negatively regulates *mycoplasma pneumonia*-triggered immune response via MAPK signaling pathway

Yuanyuan Zhang[1], Shufen Mei[1, 3], Yunlian Zhou[1], Dehua Yang[1], Ting Pan[2], Zhimin Chen[1*], Qingqing Wang[2*]

1. The Children's Hospital of Zhejiang University School of Medicine, Hangzhou 310051, P.R.China;

2. Institute of Immunology, Zhejiang University, Hangzhou 310058, P.R.China;

3. Departement of Pediatrics, Red Cross Hospital of Hangzhou, 310003; P.R.China.

*Correspondence:

Zhimin Chen, The Children's Hospital of Zhejiang University School of Medicine, No. 3333 Binsheng Road, Hangzhou 310051, P.R. China.

Tel: (+86-571)-87061007, Fax: (+86-571)-87033296. E-mail: zmchen@zju.edu.cn.

Additional correspondence: Qingqing Wang, Institute of Immunology, Zhejiang University, No. 866 Yuhangtang Road, Hangzhou 310058, P.R.China.

Tel: (+86-571)-88208284, Fax: (+86-571)-88208285. E-mail: wqq@zju.edu.cn.

Supplementary information

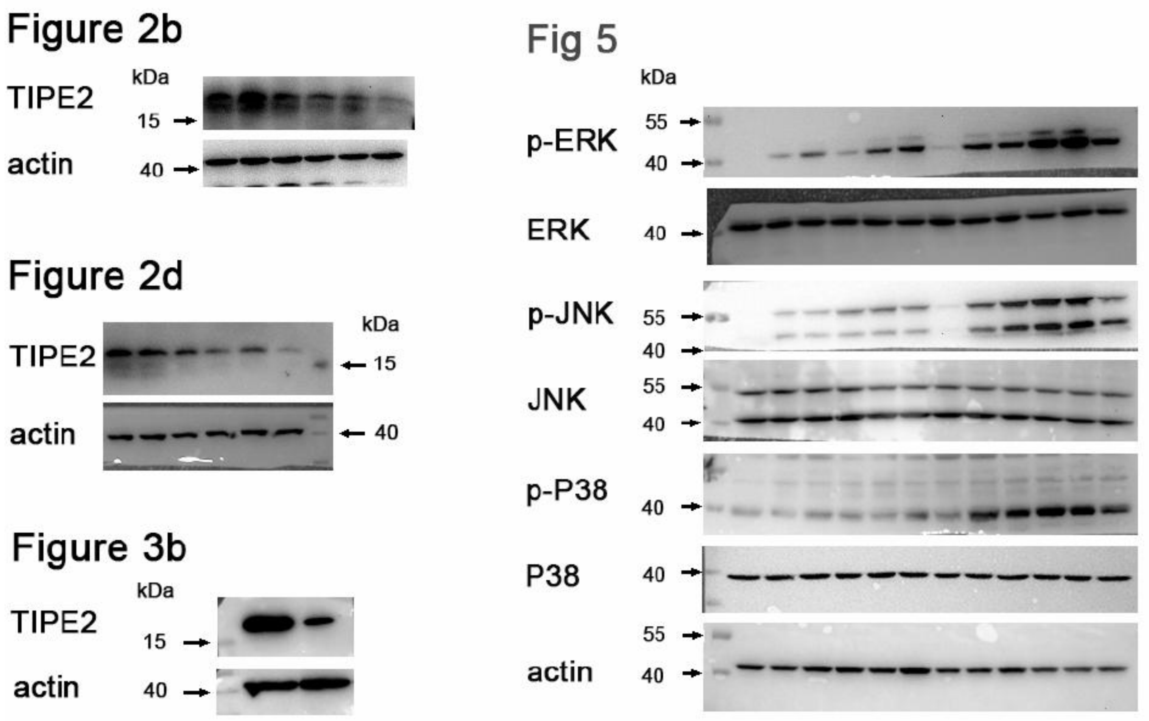

Supplement: Supplementary file 1 — Supplementary Information File [file 41598_2017_13825_MOESM1_ESM.pdf]
